# Shock Absorbing Function Study on Denucleated Intervertebral Disc with or without Hydrogel Injection through Static and Dynamic Biomechanical Tests In Vitro

**DOI:** 10.1155/2014/461724

**Published:** 2014-06-22

**Authors:** Zhiyu Zhou, Manman Gao, Fuxin Wei, Jiabi Liang, Wenbin Deng, Xuejun Dai, Guangqian Zhou, Xuenong Zou

**Affiliations:** ^1^The Medical School of Shenzhen University, Shenzhen 518061, China; ^2^Department of Spinal Surgery/Orthopaedic Research Institute, The First Affiliated Hospital of Sun Yat-sen University, Guangzhou 510080, China; ^3^Pharmacy Department, The Fifth Affiliated Hospital of Sun Yat-sen University, Zhuhai 519000, China; ^4^Department of Orthopaedics, The Second Affiliated Hospital of Nanchang University, Nanchang 330031, China

## Abstract

Hydrogel injection has been recently proposed as a novel therapy for disc degenerative diseases, with the potential to restore the spine motion and the intervertebral disc height. However, it remains unknown whether the new technique could also maintain the shock absorbing property of the treated intervertebral disc. In this study, 18 porcine lumbar bone-disc-bone specimens were collected and randomly divided into three groups: the normal with intact intervertebral discs, the mimic for the injection of disulfide cross-linked hyaluronan hydrogels following discectomy, and the control disc with discectomy only. In the static compression test, specimens in the mimic group exhibited displacements similar to those in the normal discs, whereas the control group showed a significantly larger displacement range in the first two steps (*P* < 0.05). With the frequency increasing, all specimens generally displayed an increasing storage modulus, decreasing loss modulus, and tan*δ*. At any frequency point, the control group exhibited the largest value in all the three parameters among three groups while the normal group was the lowest, with the mimic group being mostly close to the normal group. Therefore, the hydrogel injection into the intervertebral discs greatly restored their shock absorbing function, suggesting that the technique could serve as an effective approach to maintaining biomechanical properties of the degenerative intervertebral disc.

## 1. Introduction

Intervertebral discs, located between two vertebral bodies, are mainly composed of endplate, nucleus pulposus, and annulus fibrosus, which play a central role in permitting motion, allowing spinal flexibility, and dissipating energy during activities of daily living [1]. Compared with the annulus fibrosus composed of layers of collagen fiber lamellae, organized into centric rings around the nucleus pulposus [2, 3], the nucleus pulposus is normally hydrated and principally composed of water in a matrix of proteoglycan and other matrix proteins, appearing as observed translucent and gel-like [4, 5]. The nucleus pulposus and the annulus fibrosus are integrated together to maintain the normal biomechanical functions of the disc.

Disc degeneration is a common process that can result in degenerative disc diseases with low back pain and affect millions of people [2, 6, 7]. Disc degeneration is generally initiated from morphological and compositional changes of the nucleus pulposus [8]. Nucleus discectomy is therefore a common surgery for patients having herniated nucleus pulposus [4]. Through this surgery, most patients have their pain relieved [9]. However, increasing investigations have pointed out that nucleus discectomy greatly affects spinal structure and biomechanical functions, probably leading to further degeneration of the adjacent discs and facet joint [9, 10].

So as to overcome limitations of the current surgical treatment, it is necessary to develop products and techniques for nucleus replacement to restore the normal function of the degenerative disc. Hydrogel injection has been developed in recent years [11–14]. The hydrogel made of hyaluronic acid and/or collagen-hyaluronan has been used for injection and proven to be effective to restore the range of spine motion and the height of intervertebral space [13–15]. As one of the most important functions of the intervertebral disc, cushion and distribution of compression load (shock absorbing function), however, has been overlooked in most current studies. Appropriate shock absorption is critical for protecting the nervous system, such as brain and spinal cord.

In this study, we prepared a type of hydrogel by combining hyaluronic acid and other extracellular matrix materials. This composite hydrogel can cross-link in situ and is therefore an ideal material for injection as a nucleus replacement. In an attempt to understand the function reconstruction (especially the function of shock absorbing) of degenerative disc by hydrogel injection, we investigated the static and dynamic biomechanical characteristics of lumbar intervertebral disc with or without hydrogel injection after nucleus discectomy.

## 2. Materials and Methods

### 2.1. Materials

All materials and reagents used in this study were purchased from Sigma-Aldrich except that the hyaluronic acid (HA, Mw = 0.26 MDa, kinetic viscosity = 5 mm^2^/s, and intrinsic viscosity = 6.1 dL/g) was purchased from Bloomage Freda Biopharm Co., Ltd.

### 2.2. Hydrogel Preparation

The hydrogel was disulfide cross-linked from thiol-modified HA. Synthesis of the thiol-modified HA derivative followed exactly the same methods from a previous study [16]. There was 50–60% of carboxyl groups of HA that were transformed to thiol groups. The thiol groups could be oxidized in air to form disulfide linkages and further treated with diluted H_2_O_2_ to form additional disulfide linkages. The diluted H_2_O_2_ was added to thiol-modified HA immediately before injection into the intervertebral disc. Other extracellular matrix materials, including collagen II and chondroitin sulfate, could be premixed with the hydrogel.

### 2.3. Specimen Preparation

**Figure 1:**
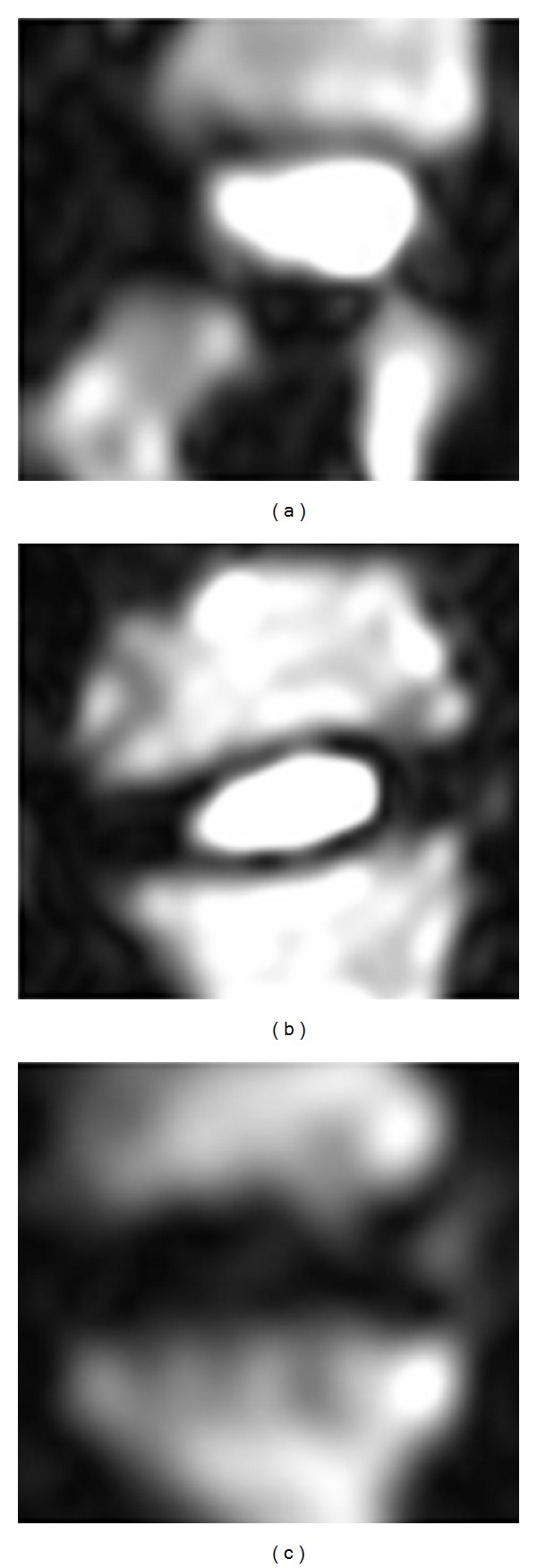
MRI examining of bone-disc-bone specimens. (a) The discectomied discs injected with hydrogel (mimic group). (b) The normal discs (normal groups). (c) The discectomied discs (control group).

Three fresh frozen spines (L1-S1) from pigs were used in this study. All methods and procedures were peer reviewed and approved by the Institutional Review Board and Ethic Committee of the First Affiliated Hospital of Sun Yat-sen University (number 2008-55). MRI examining was used to validate intact of the spines (Figure 1). All ligaments of lumbar spines were stripped off except anterior and posterior ligaments around each of lumbar intervertebral discs. Each specimen was initially made out by cutting off horizontally in the medium of the adjacent vertebra. All the bone-disc-bone specimens were randomized into normal group, mimic group, and control group. Six specimens in normal group possessed relatively intact lumbar vertebral discs without any other operation. By contrast, specimens in the other two groups were subjected to subsequent operations. At the first step, the bone-disc-bone specimens in the mimic and control groups were drilled a hole at the center of the superior surface of specimen with a diameter of 1.5 mm. Then a hooked needle was inserted through the hole and the nucleus pulposus was mashed up softly avoiding the damage of annulus fibrosus. All fragments of nucleus pulposus were washed out completely with validation of MRI examining. 1.0 mL hydrogel material was injected in each disc of mimic group (Figure 1).

### 2.4. Biomechanical Tests

**Figure 2:**
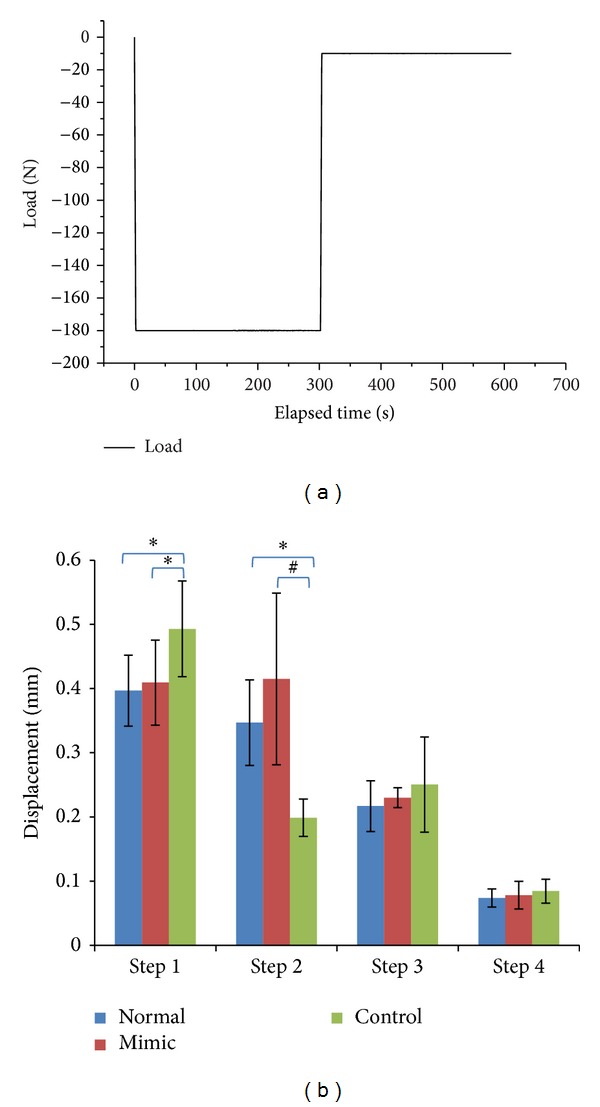
(a) Procedures of the static compression test. (b) Displacement in each step of different groups in the static compression test (*P < 0.05, ^#^ *P* < 0.01).

The biomechanical properties of each specimen were studied via static compression test and dynamic compression test, utilizing the instrument Bose ElectroForce which had been validated on the accuracy as well as precision [16]. The subsequence of static and dynamic tests was arranged according to random number. A procedure of static compression test was set up primarily including 4 steps as follows: step 1: the compression load was increased from 0 to 180 N in 2 seconds; step 2: the compression load was kept at 180 N for 5 minutes; step 3: the compression load decreased to 10 N in 2 seconds; and step 4: the compression load was kept at 10 N for 5 minutes (Figure 2(a)). The loading and displacement at each step were recorded by Bose ElectroForce and the histogram was shown in Figure 2(b). Similarly, a procedure of dynamic compression test was set up as well (i.e., at each frequency from 0.5 to 5.5 Hz, the cyclic compression load was set with median of 150 N and amplitude of 50 N and the test was sustained for 8 minutes). During the dynamic test, constant displacement of each specimen was recorded and series of mechanical parameters were exported by Bose ElectroForce including storage modulus, loss modulus, and tan*δ*.

The maximum compressive load of 180–200 N was selected to represent the human body weight scaled for differences in cross-sectional area of the human and porcine intervertebral discs [17, 18]. The minimum compressive load was 10 N because the disc of human body was still compressed even in a horizontal position [18]. The maximum frequency of 5.5 Hz was set to represent the frequency of how often the intervertebral disc was compressed by body weight when a human runs very fast (400–600 meters/minute). During the experiments, a saline humidifier was used to keep the specimens moist. And the room temperature was kept at 20–22°C. The specimens were definitely preserved in PBS 4°C for 72 h after every test to make sure that it had been recovered for the next test.

### 2.5. Statistical Analyses

All data were expressed as mean ± SEM. Data from the three groups in the static compression test were analyzed statistically using a one-way ANOVA. Univariate analysis of variance was used to statistically analyze data of each group in the dynamic compression test at different frequencies. All statistical tests were performed with SPSS 13.0 and *P* < 0.05 was considered to be significant.

## 3. Results

In step 1 of the static compression test, when the compression load was increased from 0 N to 180 N in 2 seconds, specimens in the control group exhibited significantly more displacement compared with the other two groups (*P* < 0.05), while there was no significant difference between the normal group and the mimic group (*P* > 0.05). In step 2, all specimens were continuously kept 180 N compressed for 5 minutes and values of displacement in the normal group and in the mimic group were significantly higher than those in the control group (*P* < 0.01 and *P* < 0.05, resp.), while there was not any significant difference between the normal group and the mimic group (*P* > 0.05). However, either in step 3 when the load decreased from 180 N to 10 N in 2 seconds or in step 4 when 10 N was kept to the end of test, no significant difference was found between any two groups (*P* > 0.05).

**Figure 3:**
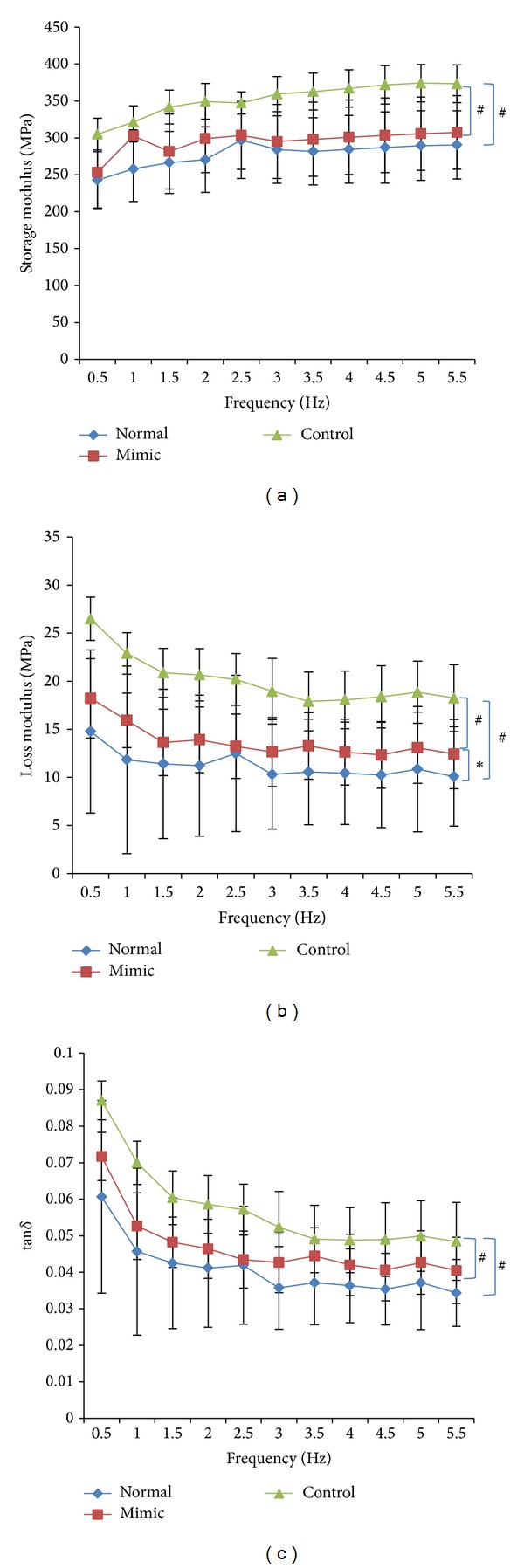
Results of the dynamic compression test. With the frequency increasing, the storage modulus increased significantly (*P* < 0.01) and both the loss modulus and tan*δ* decreased significantly (*P* < 0.01). (a) Storage modulus of control group was significantly higher than the other two groups (*P* < 0.01). (b) Loss modulus of control group was significantly higher than the other two groups (*P* < 0.01). (c) tan*δ* of control group was significantly higher than the other two groups (*P* < 0.01).

In the dynamic compression test, series of mechanical parameters were received and we chose storage modulus, loss modulus, and tan*δ* (*δ* represents phase angle) to analyze the viscoelasticity of specimens [19]. The storage modulus, loss modulus, and tan*δ* were mainly determined by both the materials per se and the frequency. Values of each parameter at 11 selected frequencies ranging from 0.5 HZ to 5.5 HZ were exhibited as curve graphs (Figure 3). As shown, values of the loss modulus were much lower than those of storage modulus in all groups. With frequency increasing, the storage modulus increased significantly (*P* < 0.01), and both the loss modulus and the tan*δ* decreased significantly (*P* < 0.01). The storage modulus, as well as the loss modulus and tan*δ* in the control group, was significantly higher than the other two groups (*P* < 0.01). The mimic group exhibited similar storage modulus and tan*δ* with the normal group (*P* > 0.05), while the loss modulus in the mimic group was significantly higher than the normal group (*P* < 0.05).

## 4. Discussion

The nucleus pulposus of the intervertebral disc could cushion and distribute the compression load to the surrounding annulus fibrosus under circumferential tension [4, 20]. Discectomy, the current surgical procedure to cut the nucleus pulposus off, often leads to disc dysfunctions. The development of the nucleus replacement could provide a promising way to break through the present clinical limitations. We compared the biomechanical properties among the normal discs (normal groups), the denucleated discs (control group), and the denucleated discs with hydrogel injection (mimic group). We found that the hydrogel injection in the mimic group could almost restore shock absorbing function of the discs.

How to restore the disc biomechanical properties of the degenerative disc has been always the focus in the study of the degenerative lumbar spine. Eyholzer et al. synthesized one kind of photoreactive nanomaterials, which could mimic the swelling and mechanical behavior of the native human nucleus pulposus [21]. Vernengo et al. verified that poly(N-isopropylacrylamide) could improve elasticity and meet the minimum stiffness of 50 kPa for the restoration of intervertebral disc stiffness in the presence of a small amount of poly(ethylene)glycol dimethacrylate [22]. However, these studies only tested the mechanical properties of nucleus and biomaterials, while these materials were not injected into the intervertebral space for mechanical test to evaluate how the disc biomechanical properties improved.

Using porcine spinal motion segments, Balkovec et al. showed that the injectable hydrogel is able to restore the height of the intervertebral space, angular stiffness to cyclically fatigued spinal motion segments [12]. Through thirteen human cadaver lumbar anterior column units, Cannella et al. [23] found that intervertebral disc instability, evidenced by increased neutral zone and ranges of motion, associated with degeneration, can be restored by volume filling of the nucleus pulposus using a synthetic hydrogel. However, no study investigated whether the hydrogel could rebuild the shock absorbing function of the disc. In the present study, we verified that the hydrogel could restore shock absorbing function of denucleated intervertebral discs through static and dynamic biomechanical tests.

Viscoelastic behavior is the most important biomechanical characteristic of the nucleus pulposus, especially for its shock absorbing function [24–26]. As it is acknowledged, the storage modulus, loss modulus, and tan*δ* are important parameters in evaluating viscoelasticity of tissues and biomaterials. The storage modulus reflects the elasticity, the loss modulus reflects the viscosity, and tan*δ*, the ratio of the storage and loss modulus, presents the mathematical description of the dynamic experiment under strains within a sample's range of viscoelasticity [19, 27]. When the compression loads on the disc, the viscoelasticity enables the disc to absorb the energy and dissipate to surrounding tissue via shape changes. After the loading disappears, the viscoelasticity enables the disc to release energy and dissipate to surrounding tissue via recovery of shape. Therefore, the disc plays an important role in shock absorbing. In this study, the three parameters were obtained via dynamic compression test and analyzed comprehensively to evaluate the alterations in viscoelasticity of the disc with or without hydrogel injection after nucleus discectomy.

The storage modulus of specimens in the control group was statistically greater than those in the normal group (*P* < 0.01) and mimic group (*P* < 0.01), revealing that discs got stiffer after discectomy while hydrogels injection was conductive to restore the disc normal elasticity. Similarly, the loss modulus was significantly greater in the control group than in the other two groups (*P* < 0.01), suggesting that discectomy caused the disc to be more viscous and hydrogel injection could largely alter the viscosity though it was still statistically greater than the normal group (*P* < 0.05). It is acknowledged that the storage modulus and loss modulus are closely related with material properties, procession conditions, and even molecular structure. Annulus fibrosus contains more collagen fiber while nucleus pulposus is richer in proteoglycan [4, 5]. What is more, it has been proved that their viscoelastic properties showed obvious difference. Results in this study were in line with Freeman et al.' study [28]. It was found that annulus and fibrous had similar storage modulus and loss modulus, which were greater than the nucleus. Interestingly, it was demonstrated that the whole disc dynamic shear moduli was increased with aging and degeneration and the observed increase was coincident with increased fibrotic tissue [8]. Nevertheless, the annulus fibrosus changes the mode of motion after discectomy, which would also alter the disc storage and loss modulus [29, 30]. Above all, we firstly illustrate how the dynamic compressive moduli of the whole disc changed after discectomy and whether hydrogel injection could restore the normal properties.

Figure 3(c) showed that tan*δ* was statistically greater in the control group compared with the other two groups (*P* < 0.01), while it was similar between the mimic group and normal group (*P* > 0.05), suggesting that hydrogel injection contributed in improving the viscoelasticity. tan*δ* equals the ratio between the storage modulus and the loss modulus. Specimens in the control group possessed greater tan*δ*, suggesting that they were more elastic and less viscous [28]. Furthermore, tan*δ*, the lag between the displacement and the load, could be interpreted as the ratio between the energy dissipated and the energy stored, which is mainly involved in the shocks absorbing function of the disc.

It is evident in MRI images (Figure 1) that the intervertebral space in the control group was filled with air after dicectomy. Therefore, in the first step of the static compression test, displacement in the control group was larger than those in the other two groups because air in specimens of the control group was easily compressed. This result demonstrated that hydrogel injection was helpful to recover the response to rapid compression. In step 2, 180 N was continuously loaded for 5 minutes and specimens in the control group showed less displacement than those in the other two groups, which demonstrated that they were harder to be compressed. Hydrogel injection reversed the situation. Normal nucleus pulposus is viscoelastic, transmitting compression load to surrounding annulus fibrosus and maintaining the normal biomechanical properties [5, 20]. After discectomy, there was only annulus fibrosus left to support compression load, so that inner boundaries moved inwards while outer boundaries moved outwards after nucleus discectomy [29, 30]. Though there have been no investigations comparing the viscoelastic properties of the annulus fibrosus and the normal disc, it has been reported that the annulus fibrosus possessed larger modulus than the nucleus pulposus [19, 31, 32], which made the annulus fibrosus harder to compress. It is suggested that the alterations of both disc response to load and the viscoelasticity resulted in specimens that were harder compressed after discectomy. The results were coincident with those of the dynamic compression test.

The integrity of intervertebral disc is necessary for the effective disc functions [33]. These functions are highly dependent upon the integrity of the annulus fibrosus and endplates. Therefore, even though it is much easier to measure the biomechanical function of nucleus pulposus alone [19], we have established this study using bone-disc-bone specimens [4, 34, 35]. The results showed not only the effective role of the artificial nucleus pulposus, but also the possible adverse events associated with the discectomy.

One of limitations in this study was the loading on the samples lower than other studies (about 300–500 N). It is because the vertebras in this study came from the minipig (weighted about 25 kg), which weighted much lower than normal adult. Therefore, we chose 150~180 N in this study to avoid broking of the vertebras and annulus fibrosus. Another limitation in this study was that the samples came from minipigs, whose discs are a little different from human discs. However, the bone-disc-bone specimens, which possess the integrity of the endplates, annulus fibrosus, and nucleus pulposus, could satisfy the requirement of a biomechanical study [12].

## 5. Conclusion

In conclusion, discectomy greatly alters the biomechanical function of the disc, especially shock absorbing. On the other hand, hydrogel injection could largely restore the biomechanical function including shock absorbing, which is a promising clinical treatment in the future.
